# Can Lateral Cyclic Reactive Jump Ability Be Reliably Measured Using an Inertial Measurement Unit?

**DOI:** 10.3390/s26206532

**Published:** 2026-10-15

**Authors:** Margaret M. Michalak, Chloe M. Ryan, Alex Ehlert, Yuxiao Lian, Christopher Slocum, Leslie Bennett, Hailey Villarreal, John Cronin

**Affiliations:** 1 Sports Research Institute New Zealand, Health and Environmental Sciences, Auckland University of Technology, Auckland 0632, New Zealand; 2 Athlete Training and Health, Cypress, TX 77429, USA; 3 Department of Sport and Health, Faculty of Health, Education and Environment, Toi Ohomai Institute of Technology, Tauranga 3112, New Zealand; 4 Potential Performance, Tauranga 3112, New Zealand; 5 Independent Researcher, Durham, NC 27701, USA; 6 Department of Sports Medicine, University of St. Thomas, Houston, TX 77006, USA

**Keywords:** change of direction, stretch-shortening cycle, contact time, air time, female athletes, wearable technology, sports performance

## Abstract

**Highlights:**

**What are the main findings?**

- The wearable IMU demonstrated variable reliability for quantifying contact time and air time during repeated lateral reactive jumping, with no testing condition meeting both predefined absolute and relative reliability criteria.
- Measurement variability generally increased at greater lateral jump distances, while the reliability of contact time and air time differed across testing conditions.

**What are the implications of the main findings?**

- Reliability established for an IMU during conventional vertical reactive jump assessments should not be assumed to transfer to more complex, multidirectional movement tasks.
- Further refinement of the lateral cyclic jump protocol is required before IMU-derived contact time and air time can be confidently used to monitor small changes in athlete performance.

**Abstract:**

Jump testing is widely used to assess lower-limb power, force absorption, and stretch-shortening cycle (SSC) function; however, lateral cyclic tests remain underexplored despite their relevance to change-of-direction performance. This study determined the intra- and inter-session reliability of a novel cyclic lateral leg power test using an inertial measurement unit (IMU). Seventeen female athletes completed three identical testing sessions separated by seven days. Participants performed 10 consecutive lateral bounds at distances of 1.0×, 1.25×, and 1.5× leg length on each limb. A foot-mounted inertial measurement unit (IMU) (Output Sports Ltd., Dublin, Ireland) quantified contact time (CT) and air time (AT), with the five shortest values for each variable analyzed. Reliability was assessed using percent change in the mean, coefficients of variation (CV), and intraclass correlation coefficients (ICC). Between-session changes were small (−7.9% to 6.6%), with no significant session effects for CT or AT. CVs ranged from 9.2% to 21.8%, with only CT at 1.0× leg length between Sessions 1 and 2 below 10%. ICCs ranged from 0.12 to 0.76, indicating poor-to-good relative reliability. Overall, the IMU-instrumented lateral cyclic test demonstrated largely unacceptable reliability, particularly at greater jump distances, indicating that further protocol refinement is required before implementation in clinical or performance settings.

## 1. Introduction

Jumping assessments are widely used in both athletic performance testing [1,2] and physical therapy rehabilitation [3,4] as objective measures of lower-limb function. These tasks provide insight into an athlete’s ability to generate force, absorb mechanical energy, express lower-limb power, and tolerate rapid loading during dynamic movements [2,5]. Single-leg hop tests are frequently used in sport performance settings to evaluate unilateral propulsion and landing capacity and in rehabilitation and clinical settings to monitor progress and guide return-to-sport decision-making [6,7,8]. Furthermore, jump performance can be evaluated across multiple planes of movement, including vertical, horizontal, and lateral directions [9,10]. Prior to using such tests, establishing test reliability is essential before an assessment can be implemented in practice, as practitioners must be able to distinguish meaningful changes in performance from normal biological and measurement variability [11]. Observed variability between repeated measurements may reflect both natural biological variation and measurement error associated with the device and testing protocol; therefore, establishing reliability within the specific testing context is necessary to determine whether observed changes reflect meaningful differences in performance.

Vertical jumps, such as the countermovement jump (CMJ), are among the most commonly studied and have well-established validity and reliability [12,13,14], making them a standard measure of lower-limb power. High test–retest reliability has been reported for force plate-derived vertical jump assessments, with ICCs ranging from 0.82 to 0.98 and low coefficients of variation (CV) (1.7–9.4%) [14,15]. Horizontal and lateral jump assessments have been introduced to better reflect the multidirectional nature of sport movements, particularly those involving cutting and change of direction (COD) [7,16,17]. Authors of a review of direction-specific jump assessments reported generally high relative reliability (ICCs), although coefficients of variation (CV) varied across the assessments examined [9]. For example, Meylan et al. [17] reported low between-trial changes in mean (1.3–2.2%), low CVs (4.0–4.4%), and high ICCs (0.91–0.94) for a discrete single-leg lateral countermovement jump assessed using a force plate [17]. Similarly, Simpson and Cronin [18] reported high between-session reliability for force plate-derived ground contact time (GCT) during unilateral horizontal reactive jumping across approach distances corresponding to 80%, 120%, and 160% of leg length, with CVs of 4.9–6.7% and ICCs of 0.90–0.95. Importantly, reliability did not progressively deteriorate as approach distance increased. While these assessments provide important information regarding unilateral leg power, they have predominantly examined discrete lateral jumps or horizontal reactive tasks rather than repeated lateral bounds requiring successive unilateral deceleration and redirection of momentum. Consequently, their reliability may not directly translate to a cyclic lateral task. Cyclic tasks may better capture the repeated stretch shortening cycle (SSC) demands encountered in sport [10,19].

Cyclic tests involve repeated efforts on the same limb and are performed as maximal attempts, making them useful for assessing unilateral leg power, dynamic stability, and limb symmetry [6,8,19]. Cyclic reactive tests, such as the 10–5 pogo test, are used to quantify repeated fast SSC performance through consecutive vertical jumps with short GCTs [20,21]. Performance is commonly quantified using the reactive strength index (RSI), with vertical (RSIvert = jump height/GCT) incorporating both jump height and GCT to provide insight into SSC efficiency [20,21]. Excellent reliability (ICC ≥0.80, CV = 1.4–6.2%) has been reported for RSI_vert__ from the 10–5 pogo test using laboratory- and wearable- based measurement systems [13,20,22,23]. Other cyclic jumps include the triple leg hop for distance, crossover hop for distance, and 6 m hop for time, which are often utilized in rehabilitation settings for return-to-play protocols [19,24]. Fair to excellent reliability has been reported for these field-based cyclic jump assessments using manual tape-measure and stopwatch-based measurement methods (ICC = 0.66–0.97), with standard errors of measurement (SEMs) ranging from 11.2 to 28.8 cm for distance-based outcomes and 0.06–0.13 s for the 6 m timed hop [19,24,25]. In contrast, more detailed temporal characteristics of horizontal reactive performance have been quantified using video-based and other measurement systems [26]. Davey et al. used video analysis to identify touchdown and toe-off during the triple hop, reporting moderate-to-excellent within-session reliability for horizontal reactive strength index (RSIhor [ICC = 0.53–0.94]) [26]. Between-session reliability of RSIhor across previous studies has ranged from ICC = 0.55–0.95, with CVs of 4.1–12.5%. Collectively, these findings demonstrate variable relative and absolute reliability across repeated jump assessments, with reliability dependent on the specific outcome and measurement approach employed. However, existing cyclic assessments have predominantly focused on vertical reactive jumping or horizontal distance-based hopping, with comparatively little attention given to repeated lateral tasks, requiring successive unilateral braking and propulsive forces. Notably, triple hop testing has typically relied on stopwatches and measuring tapes to quantify performance and inform clearance decisions [27]; however, such measures provide limited information regarding the temporal and mechanical characteristics underpinning performance [28,29].

The measurement of the RSI usually involves advanced technology such as force plates [28,30], videography [28], or optogates [29,30], given the need to quantify flight and contact times. An alternative option to these technologies is the use of IMUs, which are being increasingly adopted to provide a more comprehensive and practical approach to movement assessment in both performance and rehabilitation settings [31,32]. IMUs are portable devices equipped with sensors that can measure and record movement and joint kinematics, providing insight into movement and performance characteristics, including inter-limb differences and propulsive and reactive performance [31,32]. Importantly, the reliability and validity of IMU-derived jump metrics have previously been examined during established reactive jump assessments. Specifically, Clancy et al. [23] reported acceptable reliability for Output Sports IMU-derived RSI during the 10–5 rebound jump test (ICC = 0.89; CV = 5.2%). Similarly, Montoro-Bombú et al. [33] reported good-to-excellent reliability for Output IMU-derived temporal and jump performance measures during the drop jump, with ICCs of 0.825 for GCT, 0.928 for air time, 0.921 for jump height, and 0.772 for RSI. However, the measurement properties established during vertical reactive tasks cannot necessarily be assumed to translate to a novel lateral cyclic task, which may introduce different biological and coordinative demands associated with repeated unilateral deceleration and redirection of momentum in the frontal plane. Establishing the reliability of IMU-derived temporal measures within this novel movement context is therefore necessary before such assessments can be incorporated into athlete monitoring or rehabilitation.

From this brief treatise of the literature, it is apparent there is limited research that has examined the reliability of repetitive lateral bounding tasks requiring successive unilateral force absorption and redirection. As the measurement of vertical GCT has been shown to represent SSC efficiency [34], a reliable test evaluating the repetitive lateral expression of leg power may therefore provide valuable insight into an athlete’s ability to manage and redirect lateral forces. Incorporating these assessments into rehabilitation and performance testing may better reflect the eccentric and decelerative demands underpinning COD performance, as the ability to reduce momentum and control body position in the lateral plane is fundamental to successful directional changes [35]. In this context, a lateral reactive strength index (RSI_lat_) may provide a useful measure of lateral SSC reactivity during cyclic tasks. Although RSIlat provides a conceptual rationale for integrating temporal and displacement characteristics of lateral reactive performance, RSIlat was not calculated in this study; rather, CT and AT were examined independently as temporal components relevant to such an assessment. Shorter GCTs may reflect a greater capacity to rapidly transition from braking to propulsion during SSC actions [36,37], which is important for rapid reacceleration during COD tasks [35], and when considered alongside jump distance and air time, may offer further insight into lateral explosiveness and reactive capacity [33,36]. Furthermore, current return-to-play protocols following anterior cruciate ligament reconstruction (ACL-R) largely focus on vertical and horizontal jump tasks, leaving lateral decelerative and explosive capacities comparatively underassessed [38,39,40]. A cyclic lateral jump test may therefore serve as an indicator of these qualities, offering a practical measure of lateral power expression under reactive conditions. Given this preamble, the purpose of this study was to determine the between-session reliability of IMU-derived CT and AT during a novel cyclic lateral leg power test and establish the measurement consistency of these temporal variables when the wearable technology is applied to repeated lateral reactive jumping.

## 2. Materials and Methods

### 2.1. Experimental Approach to the Problem

All participants completed three identical testing sessions separated by a period of 7 days. The same tester administered all three sessions for the assigned group, from which between-session reliability was quantified, as measured by a percent change in the mean, coefficient of variation (CV) and intraclass correlation coefficient (ICC). The variables of interest were: (1) AT; and (2) CT.

### 2.2. Participants

Participants included 18 university-level female soccer athletes. One participant withdrew following the first testing session and was excluded from analysis, resulting in a final sample of 17 participants (age: 19.5 ± 0.98 years; height: 162.1 ± 5.9 cm; body mass: 59.6 ± 6.8 kg; leg length: 84.6 ± 4.3 cm). Female athletes were specifically recruited given the relative lack of research examining lateral power and reactive strength qualities in this population, while university soccer athletes were chosen based on the availability and accessibility of a local university team. Dominant limb was self-reported prior to the first testing session, with 13 participants identifying as right-leg dominant and 4 as left-leg dominant. Participants were free of lower-limb musculoskeletal injuries in the preceding six months and currently involved in training or sport participation at least three times per week. All participants provided written informed consent prior to participation, and the study was approved by the institution’s ethics review board (AUTEC 25/226). A sample size estimate based on Borg et al. [11] was conducted to determine the number of participants required to assess the reliability of the methods. Based on a minimum acceptable reliability of 0.7 and an expected reliability of 0.9, with an assurance probability of 80% and a significance level of 0.05, 17 participants were estimated to be an adequate sample size.

### 2.3. Equipment

**Figure 1:**
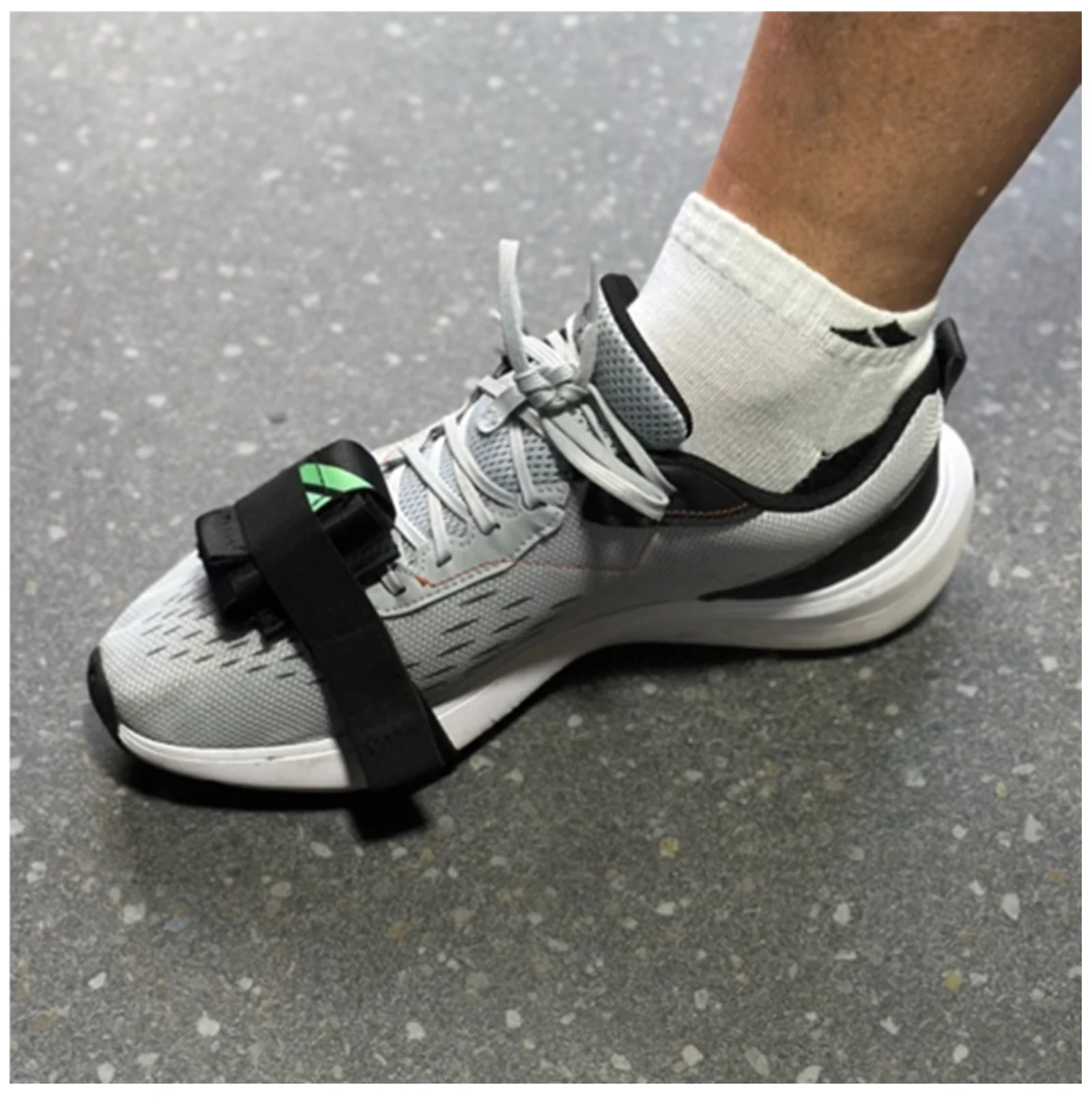
Location of Output IMU sensor.

Inertial measurement unit (IMU): The Output Capture (Output Sports Ltd., Dublin, Ireland, Version V2) was used to measure key performance metrics. The IMU was attached to a strap worn on the participant’s foot (Figure 1) and sampled at 500 Hz. The strap was positioned over the shoelaces and fastened firmly by the same examiner prior to each testing session to minimize movement of the sensor relative to the foot; footwear was standardized to lace-up athletic shoes for all participants. The wearable sensor was used to identify the temporal characteristics of each lateral bound and quantify CT and AT. The Output Sports system employs machine-learning algorithms to assess jump movements using accelerometer signals [33]. CT was defined as the interval between ground contact and subsequent take-off, whereas AT was defined as the interval between take-off and subsequent ground contact. Throughout this study, CT refers to this ground-contact phase; the term GCT is retained only when describing outcomes reported as GCT in the previous literature. These temporal events were identified automatically by the Output Sports software (version 2.55.0), with CT and AT subsequently provided for each repetition of the lateral cyclic jump test.

Tape Measure: A tape measure was used to determine leg length to the nearest cm.

### 2.4. Testing Procedures

**Figure 2:**
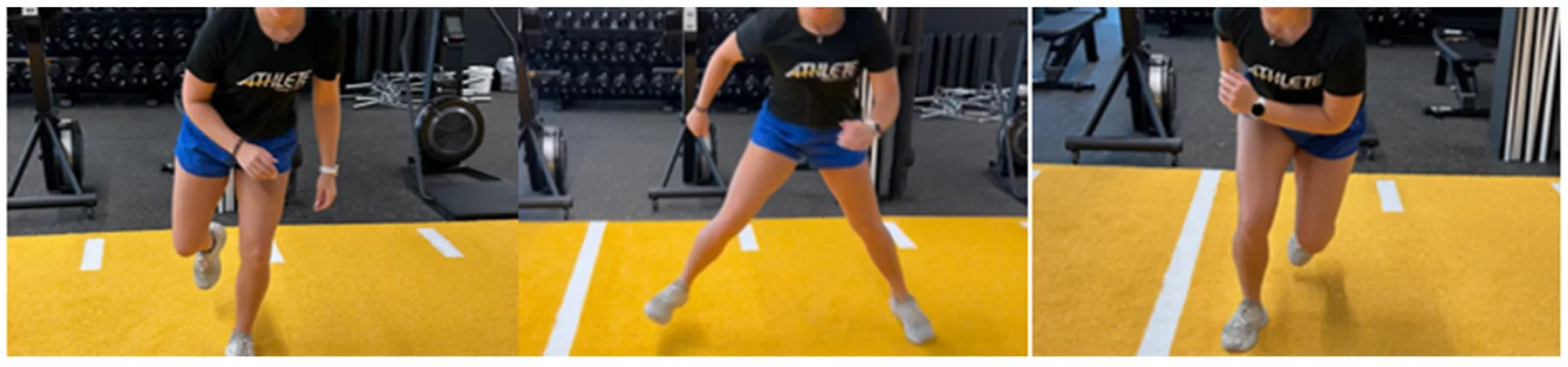
Athlete performing the lateral jump test.

Lateral Cyclic Jump Test: On arrival participants read the information sheet and signed a consent form. Participant age, height (Seca GmbH & Co. KG, Hamburg, Germany), weight (Seca GmbH & Co. KG, Hamburg, Germany), dominant leg, sporting training history and leg length from the anterior superior iliac spine (ASIS) to the medial malleolus were measured. The dominant leg was reported as the leg that participants kicked with. At each testing session, participants performed a standardized dynamic warm-up consisting of jogging and dynamic exercises, followed by familiarization repetitions of the lateral leg power assessments (Table 1). The lateral bound test was performed alternately on both legs. Participants began each trial from a single-leg stance positioned at a distance of 1.0× their leg length from a marker cone. Leg length was measured using a tape measure, and the corresponding 1.0×, 1.25×, and 1.5× leg-length distances were measured along the floor. Each target distance was marked with tape on the testing surface, with a cone positioned adjacent to the tape marker to provide a clear visual reference while minimizing the risk of participants contacting the cone during testing. From this position, they performed a lateral bound toward the cone and immediately rebounded back to the starting point, with instructions to complete the out-and-back movement (Figure 2) as quickly as possible while maintaining control for 10 repetitions. Participants performed each lateral bound as explosively as possible, aiming to cover the required lateral distance while minimizing ground contact time. They were asked to maintain upright trunk posture, land with the whole foot contacting the ground (not just the toes or heel), and stabilize briefly if needed before initiating the next jump. Brief stabilization while maintaining unilateral stance was permitted and the resulting CT was retained, as this formed part of the participant’s natural execution of the task. Trials involving loss of balance, contralateral foot contact, or other predefined technique violations were discarded and repeated following the prescribed rest period. Adequate control was monitored by observing: no excessive trunk lean or rotation during landing or take-off; no significant loss of balance (e.g., needing to touch down with the opposite foot or using the arms for stability in a compensatory manner); and consistent lateral movement direction (i.e., no unintended forward/backward displacement). Any trial where these criteria were not met was repeated after a two-minute rest to ensure valid data capture. Across all testing sessions, only three trials required repetition due to failure to meet these movement-quality criteria. Sixty seconds of rest was taken between left and right lateral leg testing, whereas two minutes of rest was provided between changes in movement distance (1.0×, 1.25× and 1.5×). Prior to the first testing session, the starting leg and order of movement distances (1.0×, 1.25×, and 1.5× leg length) were determined for each participant using a random number generator, and the assigned order was maintained across all three testing sessions. These testing procedures were repeated on three occasions, separated by seven days. All sessions occurred at the same time of day, and participants were instructed to replicate their nutrition, sleep, and training routines prior to the test days. Testing was conducted by the same examiner using a standardized verbal script to ensure consistency in instructions and encouragement; specifically, the participants were instructed to “jump laterally from line to line, as fast as possible.”

### 2.5. Data Analysis

For each repetition, temporal events were automatically identified by the Output Sports software from the IMU accelerometer signal. CT was calculated as the interval between initial ground contact and subsequent take-off, while AT was calculated as the interval between take-off and the subsequent ground contact. Event detection and calculation of these temporal variables were performed automatically by the proprietary Output Sports algorithm rather than manually defined by the researchers. A repeated-measures analysis of variance (RM-ANOVA) showed no significant effect of limb for [CT; AT: F = 2.10, df = 1.56, *p* = 0.15, n^2^ = 0.002; F = 0.79, df = 1.83, *p* = 0.45, n^2^ = 0.001], and repetitions from both limbs were therefore pooled, resulting in 20 repetitions per movement distance. Pooling was applied only at the stage of repetition selection: for each participant, the five best repetitions for each variable were selected from this combined pool of 20 and averaged, meaning each participant contributed a single CT value and a single AT value to the subsequent reliability analysis. Limb was therefore not treated as an independent factor, and no additional independent observations per participant were introduced by combining the two limbs. Specifically, the five repetitions with the shortest CT were averaged to derive the CT value, while the five repetitions with the shortest AT were averaged separately to derive the AT value. Selecting the shortest repetitions independently for each variable was intended to characterize the participant’s best performance for each temporal component, rather than assuming that the same repetitions would represent optimal CT and AT performance. This approach was consistent with the selection of best repetitions commonly used in reactive strength assessments such as the 10–5 pogo test, which has been validated in previous work [13,20,22,23]. This best-performance approach was selected to characterize the participant’s maximal reactive performance capacity rather than their typical performance across all repetitions. However, selecting the best repetitions may preferentially retain extreme performances and should therefore be considered when interpreting the reliability estimates. Because CT and AT repetitions were selected independently, the repetitions contributing to the averaged CT and AT values were not necessarily the same repetitions or derived from the same limb. Accordingly, these independently selected values should not be interpreted as paired biomechanical observations from the same jump.

### 2.6. Statistical Analysis

Statistical analysis was conducted using JASP software (Version 0.18.3) [41]. Normality was assessed using Shapiro–Wilk tests in conjunction with visual inspection of Q–Q plots and histograms. Potential outliers were assessed through visual inspection of the raw data, participant means, and box plots. Descriptive statistics (mean ± SD) were reported for all variables. Percent change in mean was used to assess systematic bias between testing sessions. RMANOVA with Bonferroni-adjusted post hoc comparisons were used to determine whether systematic differences in group means were significant across testing sessions; the absence of a significant session effect was not interpreted as evidence of acceptable reliability. Sphericity was assessed using Mauchly’s test, with Greenhouse–Geisser corrections applied when the assumption of sphericity was violated. Pairwise comparisons between consecutive testing sessions (Session 2–1 and Session 3–2), including calculation of the coefficient of variation, were performed using the reliability spreadsheet developed by Hopkins [42]. To account for potential heteroscedasticity and to express measurement error in relative rather than absolute terms, data were log-transformed prior to the reliability analysis. Log transformation was undertaken because the magnitude of measurement error was expected to be proportional to the magnitude of the measured variable, such that percentage or ratio error was considered more appropriate than absolute error. Each observation was transformed using 100 × log(value), and reliability statistics were subsequently calculated on the transformed scale. The resulting error estimates were back-transformed and expressed as coefficients of variation (CV) or multiplicative error factors, thereby providing estimates of typical measurement error as a percentage of the measured value. In accordance with COSMIN (Consensus based Standards for the selection of Health Measurement Instruments) recommendations, relative reliability was assessed using intraclass correlation coefficients (ICC [1,3]), representing a two-way mixed-effects, single-measure consistency model, for pairwise comparisons between consecutive testing sessions. The ICCs were classified as follows: ‘poor’ (<0.50), ‘moderate’ (0.50–0.75), ‘good’ (0.75–0.90), and ‘excellent’ (>0.90) [5,43]. Absolute reliability/measurement error was evaluated using the coefficient of variation (CV). A CV <10% was used as a practical benchmark for acceptable measurement error, consistent with recommendations commonly applied in sports performance reliability research [42]. This threshold was considered alongside relative reliability rather than as an isolated indicator of measurement quality. Statistical significance for inferential analyses was set at α = 0.05, and 95% confidence intervals were reported for ICC estimates.

## 3. Results

Descriptive statistics for CT and AT across all sessions and distances are presented in Table 2. Values for CT ranged from 0.31 ± 0.05 s to 0.43 ± 0.17 s across all testing conditions, while AT values ranged from 0.49 ± 0.02 s to 0.65 ± 0.13 s. Variability generally increased with jump distance, with the largest standard deviations consistently observed at 1.5× leg length. Percent change in the mean ranged from −7.9% to 6.6% across all variables, distances, and session comparisons. Changes were not consistently directional across distances for CT, although the greatest increases between Session 2–1 and Session 3–2 were observed at 1.5× (−2.1 to 6.6%). In contrast, a more consistent pattern of decline across sessions, regardless of distance, was shown for AT. Despite these small fluctuations, there was no significant effect of Session for either CT (*F* (2, 32) = 0.80, *p* = 0.438, η^2^ = 0.012) or AT (*F* (2, 32) = 1.67, *p* = 0.205, η^2^ = 0.024).

Only Session 2–1 CT at 1.0× achieved a CV below 10%, whereas all remaining variables exceeded this threshold (13.1–21.8%). Measurement variability generally increased with jump distance, with the largest CV values observed at 1.5× (averaged CV = 16.0%). A significant main effect of Distance was observed for both CT (*F* (1.16, 18.62) = 18.94, *p* < 0.001, η^2^ = 0.279) and AT (*F* (2, 32) = 30.17, *p* < 0.001, η^2^ = 0.397), consistent with the observed increase in mean values at greater jump distances. The Session × Distance interaction was non-significant for both CT (*F* (2.08, 33.30) = 1.73, *p* = 0.192, η^2^ = 0.022) and AT (*F* (2.58, 41.29) = 1.24, *p* = 0.305, η^2^ = 0.010), indicating that this distance-related pattern was consistent across testing sessions. Greater variability was also evident between Sessions 2 and 3 (averaged CV = 16.2%) compared to Sessions 1 and 2 (averaged CV = 12.4%). Across variables, larger CV values were found with CT (averaged CV = 16.2%) as compared to AT (averaged CV = 12.4%).

ICC values ranged from 0.12 to 0.76 across all variables, distances, and session comparisons. Relative reliability generally improved as jump distance increased, with the strongest reliability observed at the 1.25× (averaged ICC = 0.72) and 1.5× (averaged ICC = 0.72) distances. Moderate reliability was mostly observed for CT across conditions, with ICC values remaining relatively consistent across distances and sessions (0.67–0.75), aside from Session 3–2 at 1.0× being low (0.32). In contrast, poor reliability at 1.0× but moderate-to-good reliability at 1.25× and 1.5× was found for AT. A mixed pattern of relative reliability was observed across sessions. Although the mean ICC was marginally higher between Sessions 1 and 2 (0.61) than Sessions 2 and 3 (0.56), four of six individual conditions exhibited higher ICCs between Sessions 2 and 3.

**Figure 3:**
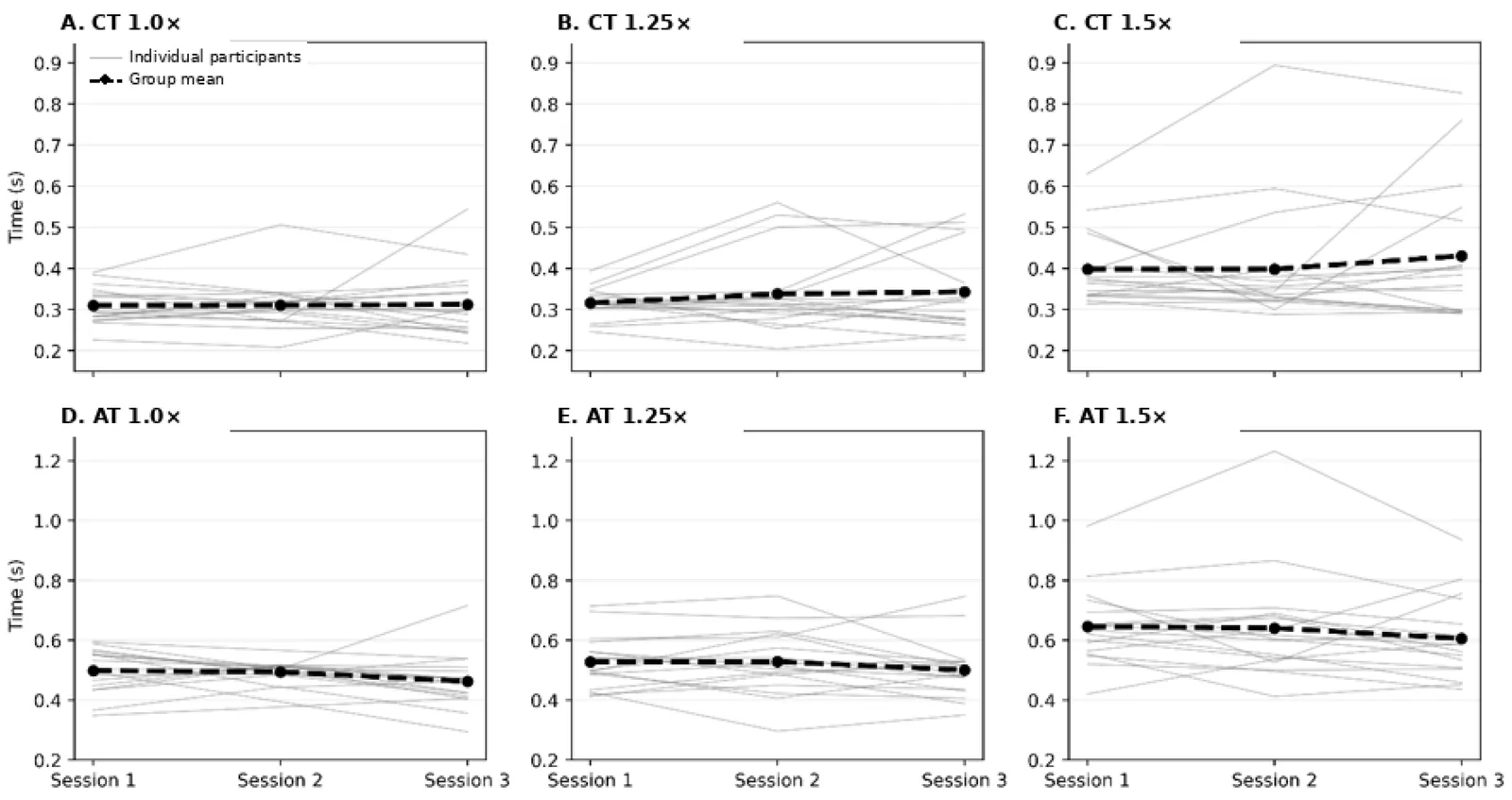
Individual participant responses for contact time (CT) (**A**–**C**) and air time (AT) (**D**–**F**) across three testing sessions at movement distances of 1.0×, 1.25×, and 1.5× leg length. Thin gray lines represent individual participants (*n* = 17), whereas the dashed black line with markers represents the group mean.

Individual participant responses across the three testing sessions are presented in Figure 3. Group mean values remained relatively stable across repeated testing sessions for both CT and AT, consistent with the absence of significant between-session differences. However, greater inter-individual variability was evident at the longer movement distances, particularly at 1.5× leg length. In contrast, participant responses for AT at the 1.0× distance were more tightly clustered, indicating that relatively small changes in performance could alter participant rank order between sessions.

## 4. Discussion

Given the importance of lateral leg power for the deceleration and propulsion demands of COD performance, establishing reliable methods to assess this quality is fundamental to monitoring performance and informing exercise prescription. Therefore, the purpose of this study was to examine the between-session reliability of IMU-derived CT and AT during a novel cyclic lateral leg power test and establish the measurement consistency of these temporal variables when the wearable technology was applied to repeated lateral reactive jumping. The principal findings were that: (1) changes in mean performance ranged from −7.9% to 6.6%, with no significant between-session differences observed for CT or AT; (2) absolute reliability, as quantified by the CV, ranged from 9.2% to 21.8%, with only CT at the 1.0× distance between Sessions 1 and 2 having a CV below 10%; and, (3) relative reliability, as quantified by ICC, ranged from 0.12 to 0.76, with CT generally demonstrating moderate reliability and AT exhibiting a more distance-dependent pattern. The selection of the five shortest repetitions independently for CT and AT was intended to characterize best performance for each temporal component rather than typical performance across all repetitions. In summary, when absolute and relative reliability were considered together, no testing condition was found to have acceptable reliability. Marginal reliability was observed for CT at the 1.0× distance between Sessions 1 and 2 and for AT at the 1.5× distance between Sessions 2 and 3, whereas all remaining conditions were found to have unacceptable reliability.

Changes in the mean ranged from −7.9% to 6.6% across variables, distances, and session comparisons. CT remained comparatively stable across sessions, with no consistent directional change across movement distances, whereas AT was found to have a greater tendency to decline across sessions. Between-session fluctuations were generally smallest at the 1.0× distance and more pronounced at the longer movement distances, particularly at 1.5×. Despite these fluctuations, no significant differences were observed for either CT or AT, indicating an absence of statistically significant systematic changes in group mean performance across the testing period. Importantly, this absence of a significant session effect should not be interpreted as evidence of acceptable measurement reliability, as demonstrated by the CV and ICC findings. The somewhat larger changes in the mean observed in the present study compared with previous lateral jump research may reflect important differences in the technology used (force plate vs. IMU) and task structure, as Meylan et al. [17] examined acyclic maximal-effort jumps without a prescribed distance, whereas the protocol used in this study required repeated unilateral lateral bounds across externally prescribed distances. The greater regulation and movement control demands of repeatedly achieving a target distance may therefore have contributed to larger between-session fluctuations, particularly at the longer distances. Although no significant systematic between-session changes were detected, this finding does not exclude the possibility of individual learning or familiarization effects. Accordingly, the absence of a separate familiarization session should be considered when interpreting the between-session reliability estimates. The individual response plots (Figure 3) further support this finding by demonstrating relatively stable group mean performance across repeated testing sessions despite modest variability in individual responses.

Absolute reliability, as quantified by the CV, varied markedly across testing conditions, with values ranging from 9.2% to 21.8%. Only CT at the 1.0× distance between Sessions 1 and 2 was observed to have a CV below the proposed 10% threshold for acceptable measurement error in sports performance testing [42]. Measurement variability was generally greater for CT than AT and tended to increase with movement distance, with the largest CVs observed at the 1.5× distance. To the authors’ knowledge no previous researchers have examined lateral leg power similar to the methods described in this paper, making direct comparisons difficult. Nevertheless, the magnitude of measurement error observed in this study is consistent with the broad variability reported across direction-specific unilateral jump assessments. Hewitt et al. summarized CVs ranging from approximately 3.3–18.2% for single-leg vertical jump tasks and 10.7–22.7% for several single-leg horizontal tasks, with no clear trend in absolute consistency across movement directions [9]. Similarly, Leidersdorf et al. reported marked between-session differences across outcomes derived from a single-leg lateral countermovement jump, assessed using dual force plates, with CVs ranging from 4.46% for peak lateral force to 22.09% for lateral rate of force development and 19.64% for total movement time [7]. Together, these findings indicate that absolute consistency in unilateral multidirectional jump testing may depend strongly on the specific outcome quantified, providing a potential explanation for the greater variability observed for CT than AT in the study. In addition, the repeated reactive nature of the protocol used in this study may have increased variability through the need to continually control and redirect lateral momentum, particularly at longer movement distances. CT may be especially sensitive to between-session differences in braking mechanics, frontal-plane stability, and force application during ground contact, whereas AT could be indirectly associated with propulsive mechanics, with greater AT indicative of greater propulsive forces. Increasing movement distance may further amplify these demands and therefore variability, given greater lateral momentum and force-absorption requirements.

Previous research has reported acceptable reliability and validity of the IMU used in this study for quantifying temporal and reactive strength variables during established vertical jump assessments [23,33]. Given these previous findings, it is difficult to determine whether the variability observed in the present study reflects technological error, biological variability, or a combination of both. However, greater variability in ground contact measures has also been reported during COD tasks. Clarke et al. reported CVs of 14.8–22.4% for GCT during the turning phase of a 505 test, compared with 2.3–6.3% for other phase-specific variables, suggesting that temporal measures associated with braking and redirection may be particularly variable [44]. Unlike predominantly vertical reactive tasks, the present assessment required participants to repeatedly attenuate and redirect lateral momentum while maintaining frontal-plane control and achieving a prescribed movement distance. These additional coordinative and neuromuscular demands provide several potential sources of within-athlete variation between sessions. Accordingly, our findings may indicate limitations in the reproducibility of the lateral cyclic testing protocol rather than limitations in the wearable IMU’s ability to quantify the underlying temporal events.

Relative reliability, as quantified by the ICC, ranged from 0.12 to 0.76 across testing conditions, indicating poor-to-good rank-order consistency between repeated sessions. However, the confidence intervals surrounding several ICC estimates were wide, indicating considerable uncertainty in the precision of these estimates. For example, an ICC of 0.67 (95% CI: 0.35 to 0.85) was observed for the AT 1.0× comparison between Sessions 1 and 2, indicating that the point estimate and its categorical classification should be interpreted cautiously. CT was generally found to have moderate relative reliability across movement distances, whereas a more distance-dependent pattern was observed for AT, with poor reliability at 1.0× but moderate-to-good reliability at 1.25× and 1.5×. A mixed pattern was also evident across session comparisons. Although the mean ICC was marginally higher between Sessions 1 and 2 than between Sessions 2 and 3, four of the six conditions were found to have higher ICCs between Sessions 2 and 3, suggesting a possible tendency toward improved rank-order consistency following the initial testing exposure. The comparatively lower ICCs observed for AT at the 1.0× distance may partly reflect the relatively homogeneous sample. Participants were all university female soccer athletes of similar age, training background, and physical characteristics, resulting in limited between-subject variability. Because ICC represents the proportion of total variance attributable to differences between individuals, reduced between-subject variability can suppress ICC values despite relatively consistent within-subject performance [43]. This pattern is visually evident in Figure 3, particularly for AT at the 1.0× distance, where participant performances were closely clustered despite small changes in rank order between sessions. Conversely, the higher ICC point estimates observed for AT at the 1.25× and 1.5× distances may reflect greater separation between athletes as task demands increased. This suggests that the relative reliability of lateral jump assessments may depend not only on movement distance but also on the specific performance characteristics being quantified. Similar outcome-dependent patterns have been reported by Leidersdorf et al., who observed substantial variation in between-session ICCs across force plate-derived variables from a lateral countermovement jump, with only four of ten outcome measures meeting their predefined reliability criteria [7]. Together, these findings suggest that relative reliability may differ according to the performance variable being assessed rather than movement direction alone. In contrast, Simpson and Cronin reported consistently high between-session reliability for force plate-derived CT across horizontal reactive jumps performed over approach distances corresponding to 80%, 120%, and 160% of leg length (ICC = 0.92–0.95) [18]. Unlike the protocol used in this study, however, their assessment consisted of isolated horizontal reactive jumps rather than repeated lateral cyclic bounds. The repeated unilateral braking, stabilization, and reacceleration demand of the lateral cyclic task may have introduced greater movement variability, particularly at longer movement distances. This pattern is also reflected in Figure 3, where the spread of individual responses becomes progressively greater at the 1.5× movement distance compared with the shorter movement distances. These contrasting findings suggest that increasing movement distance alone does not necessarily reduce relative reliability and that the influence of distance may depend on the specific structure and demands of the testing protocol.

When absolute and relative reliability were considered together, only two pairwise comparisons were found to have marginal reliability, whereas all remaining conditions were classified as unacceptable. Specifically, CT at 1.0× (Sessions 1–2) and AT at 1.5× (Sessions 2–3) met only one of the predefined reliability criteria, while no condition satisfied both acceptable ICC and CV thresholds. It appears that in its current form, the protocol lacks sufficient measurement consistency for confidently detecting small performance changes over time and therefore cannot currently be recommended as a standalone assessment for return-to-play decision-making. The absence of acceptable reliability for the lateral jump protocol used in this study is consistent with previous research indicating that measurement consistency may decrease as movement complexity increases, particularly during unilateral and multiplanar tasks [17,45]. Accordingly, the observed measurement variability may reflect a combination of task complexity, biological variability, and technological measurement error, although the relative contribution of each cannot be disentangled given the design of this study.

This study was not without limitations. First, participants did not complete a separate familiarization session prior to data collection; instead, familiarization repetitions were performed as part of the standardized warm-up at each testing session. While group-level mean changes between sessions were small, an individual learning effect cannot be fully excluded, and future work should examine whether a dedicated familiarization session, completed independently of the warm-up, improves test–retest reliability of this lateral cyclic jump protocol. As no criterion measure (e.g., force plate) was collected concurrently during the lateral cyclic task, the present findings establish repeatability of the IMU-derived CT and AT measures within this protocol but do not establish their criterion validity. Furthermore, temporal event detection was performed using a proprietary Output Sports algorithm. Additionally, although the IMU strap was fitted firmly by the same examiner across testing sessions, strap tightness was not quantitatively standardized; therefore, residual movement of the sensor relative to the foot cannot be excluded as a potential source of measurement error. Although CT and AT event definitions and the sensor sampling frequency are reported, the underlying event-detection algorithm is not publicly available, limiting complete methodological reproduction. Additionally, the algorithm has not, to the authors’ knowledge, been independently validated against a criterion measure during the lateral cyclic task used in this study. The sample tested consisted exclusively of collegiate female soccer players, limiting the generalizability of the findings to male athletes or athletes from other sports. Findings may also not extend to clinical populations, such as individuals recovering from lower-limb injury (e.g., ACL reconstruction), for whom movement strategies and reliability characteristics of a lateral cyclic jumping task may differ; future research should therefore verify these findings in more heterogeneous cohorts. Although the final sample met the a priori sample-size requirement, the observed ICCs were generally lower than the expected ICC of 0.90 used in the sample-size calculation, resulting in wide confidence intervals for several estimates and reduced precision in estimating the true reliability of these conditions. Furthermore, the greater variability observed at the longer jump distances may indicate that these conditions exceeded the optimal challenge point for some participants, potentially influencing movement consistency and reliability outcomes. More broadly, the reliability estimates reported in this study are specific to the present population, lateral cyclic movement task, testing protocol, and IMU-based measurement approach. Accordingly, these findings should not be assumed to generalize to other athlete populations, sports, movement tasks, or rehabilitation settings without further investigation. Finally, the methodological design used cannot fully disentangle the sources of the observed measurement variability, as the high CVs and low-to-moderate ICCs may reflect IMU hardware error, algorithmic event-detection bias, or genuine biological and movement-coordination variability inherent to lateral cyclic jumping. Future research should therefore examine larger and more diverse athlete populations and incorporate a dedicated familiarization session to further establish the reliability of this protocol.

### Practical Applications

Although IMU-derived CT and AT may provide complementary information regarding different phases of lateral jump performance, it appears that further refinement of the testing protocol is required before these measures can be confidently used for longitudinal athlete monitoring. Specifically, CT may provide insight into the braking-to-propulsion transition, whereas AT may reflect aspects of lateral propulsion. Together, these variables have the potential to provide a more comprehensive assessment of lateral leg power than either measure alone.

Given the importance of rapid braking and force redirection during sport-specific COD tasks, movement-specific assessments of lateral reactive performance remain of considerable practical interest. Progressively increasing lateral movement distance also increases the reactive overload imposed on the athlete by requiring greater attenuation of lateral momentum before rapid reacceleration in the opposite direction. These greater braking demands may expose individual differences in eccentric strength, frontal-plane control, and reactive force production that are less apparent during shorter or more constrained jump tasks. Accordingly, assessments performed over progressively greater lateral distances may provide practitioners with additional insight into an athlete’s capacity to tolerate and redirect lateral forces relevant to COD performance. However, the moderate-to-high measurement variability observed in this study limits the protocol’s ability to reliably detect small within-athlete changes over time. One potential approach for future investigation may be to establish athlete-specific baseline variability across repeated assessments and use this variability to define individualized thresholds for meaningful change. Rather than applying a single group-level reliability threshold, changes exceeding an athlete’s typical within-individual variability may be more likely to represent a true change in performance rather than measurement noise [46]. Consequently, while this assessment may have potential as a movement-specific evaluation of lateral leg power, additional refinement is required before it can be recommended for athlete monitoring, rehabilitation, or return-to-sport decision-making.

## 5. Conclusions

This study examined the between-session reliability of IMU-derived CT and AT during a novel lateral cyclic jump test in female athletes. Although group mean performance remained relatively stable across repeated testing sessions, the measurement consistency of CT and AT within the current protocol was insufficient for confidently detecting small longitudinal changes in performance. Importantly, these findings should be interpreted within the context of the established reliability of wearable IMU technology during more conventional reactive jump assessments. The variability observed in this study may reflect a combination of biological, coordinative, movement-specific, and measurement-related factors; however, the relative contribution of these sources cannot be determined from the present study design. Accordingly, the findings should be interpreted as demonstrating limited reliability of the IMU-based measurement approach within this specific lateral cyclic testing protocol, rather than as evidence that the IMU technology itself is unreliable. Further refinement and validation of the lateral cyclic protocol is warranted before its application to longitudinal athlete monitoring, rehabilitation, or return-to-sport decision-making.

## Figures and Tables

**Table 1:** Warm up exercises.

Exercise	Reps
10 m jog there and back	×4
10 m high knees + 10 m butt flicks	×2
Walking lunge and twist	×10
10 m side lunges there and back	×2

**Table 2:** Between-Session Reliability.

Variables	Mean ± SD			% Change in Mean (95% CI)		CV (95% CI)		ICC (95% CI)	
	Session 1	Session 2	Session 3	Session 2–1	Session 3–2	Session 2–1	Session 3–2	Session 2–1	Session 3–2
*1.0*									
*CT* (s)	0.31 ± 0.05	0.31 ± 0.06	0.31 ± 0.08	0.0 (−5.4–5.6)	−1.7 (−12.0–9.8)	9.2 (7.1–13.6)	19.6 (14.9–29.3)	0.75 (0.49–0.89)	0.32 (−0.11–0.65)
*AT* (s)	0.50 ± 0.07	0.49 ± 0.02	0.46 ± 0.09	−0.4 (−7.1–6.9)	−7.9 (−15.1–0.0)	12.1 (9.2–17.8)	14.1 (10.8–20.9)	0.12 (−0.31–0.51)	0.17 (−0.26–0.54)
*1.25*									
*CT* (s)	0.32 ± 0.04	0.34 ± 0.10	0.34 ± 0.10	6.3 (−1.5–14.8)	−0.2 (−9.4–9.9)	13.1 (10.0–19.3)	16.9 (12.9–25.2)	0.67 (0.35–0.85)	0.70 (0.41–0.86)
*AT* (s)	0.53 ± 0.09	0.53 ± 0.11	0.50 ± 0.10	0.5 (−5.8–7.1)	−6.2 (−12.3–0.3)	10.9 (8.3–16.0)	11.4 (8.7–16.8)	0.74 (0.48–0.88)	0.75 (0.48–0.89)
*1.5*									
*CT* (s)	0.40 ± 0.09	0.40 ± 0.15	0.43 ± 0.17	−2.1 (−10.9–7.6)	6.6 (−5.7–20.4)	16.4 (12.5–24.4)	21.8 (16.5–32.8)	0.68 (0.37–0.85)	0.67 (0.35–0.85)
*AT* (s)	0.65 ± 0.13	0.64 ± 0.19	0.61 ± 0.14	−2.2 (−9.2–5.4)	−4.3 (−11.4–3.3)	12.8 (9.8–19.0)	13.1 (10.0–19.4)	0.75 (0.48–0.89)	0.76 (0.50–0.89)

**Description.** CT = contact time (s); AT = air time (s); CV = coefficient of variation; ICC = intraclass correlation coefficient; CI = confidence interval. Mean ± SD values are presented for each testing session. Percent change in mean, CV, and ICC values were calculated from pairwise comparisons between consecutive testing sessions (Session 2–1 and Session 3–2). CV values represent the typical error expressed as a percentage. ICC values quantify relative reliability, with higher values indicating greater consistency between sessions. Values in bold indicate estimates meeting the predefined individual reliability criterion (CV < 10% or ICC ≥ 0.75). No testing condition met both the CV and ICC criteria.

## Data Availability

The data supporting the findings of this study are available from the corresponding author upon reasonable request. The data are not publicly available due to ethical and participant privacy considerations.
